# Observational Characterization of the Ecological and Environmental Features Associated with the Presence of Oropouche Virus and the Primary Vector *Culicoides paraenesis*: Data Synthesis and Systematic Review

**DOI:** 10.3390/tropicalmed6030143

**Published:** 2021-08-02

**Authors:** Christine E. S. Walsh, Michael A. Robert, Rebecca C. Christofferson

**Affiliations:** 1 Department of Pathobiological Sciences, School of Veterinary Medicine, Louisiana State University, Baton Rouge, LA 70803, USA; csmi198@lsu.edu; 2 Department of Mathematics and Applied Mathematics, Virginia Commonwealth University, Richmond, VA 23284, USA; robertma@vcu.edu

**Keywords:** Oropouche, *Culicoides paraenesis*, arbovirus, febrile illness

## Abstract

Oropouche virus (OROV), a member of the *Orthobunyavirus* genus, is an arthropod-borne virus (arbovirus) and is the etiologic agent of human and animal disease. The primary vector of OROV is presumed to be the biting midge, *Culicoides paraenesis,* though *Culex quinquefasciatus*, *Cq. venezuelensis*, and *Aedes serratus* mosquitoes are considered secondary vectors. The objective of this systematic review is to characterize locations where OROV and/or its primary vector have been detected. Synthesis of known data through review of published literature regarding OROV and vectors was carried out through two independent searches: one search targeted to OROV, and another targeted towards the primary vector. A total of 911 records were returned, but only 90 (9.9%) articles satisfied all inclusion criteria. When locations were characterized, some common features were noted more frequently than others, though no one characteristic was significantly associated with presence of OROV using a logistic classification model. In a separate correlation analysis, vector presence was significantly correlated only with the presence of restingas. The lack of significant relationships is likely due to the paucity of data regarding OROV and its eco-epidemiology and highlights the importance of continued focus on characterizing this and other neglected tropical diseases.

## 1. Introduction

The *Orthobunyavirus* genus, which consists of 18 serogroups, has been isolated from numerous countries in South America, most commonly Brazil [1,2,3,4,5,6,7,8,9]. The largest serogroup in the *Orthobunyavirus* genus, the Simbu sergroup, includes viruses associated with both human and animal diseases and is comprised of 25 viruses, including Oropouche virus (OROV) [10,11]. OROV is an enveloped, negative-sense, single-stranded RNA virus, and the genome consists of three separate segments: small (S), medium (M), and large (L). These S, M, and L segments encode for the nucleocapsid protein, surface glycoproteins (Gn and Gc), and the RNA-dependent RNA polymerase, respectively [12]. Additionally, the OROV N gene encodes for non-structural proteins NSs and NSm [13,14].

OROV has been associated with a human febrile illness with generalized symptoms such as fever, headache, and myalgia [7,15,16,17]. The first known case of Oropouche fever was described in Trinidad in 1955. The patient was a young male, whose occupation was to burn coal in the Melajo Forest, where he often slept [4]. Neutralizing antibodies were detected in blood specimens from forest workers in various forested sites in Trinidad. Neutralizing antibodies were also found in serum samples of 7/52 monkeys throughout the area [4]. Since then, there have been outbreaks of Oropouche Fever in Brazil, Ecuador, and Peru [8,17,18]. In the past two decades, there has been progress in developing diagnostic tools for OROV and understanding of the pathogenesis of this virus [19,20,21,22].

**Figure 1:**
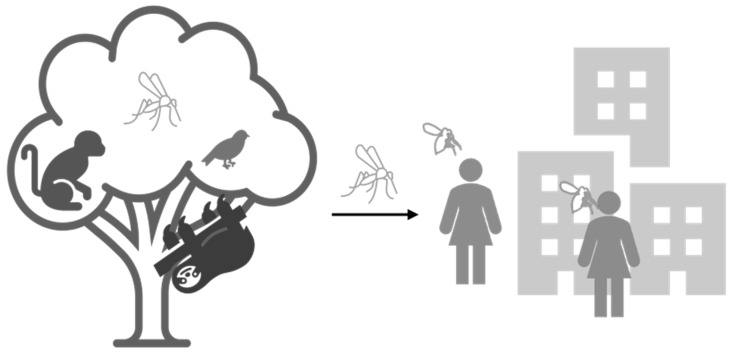
OROV transmission cycles as currently understood. In the sylvatic cycle, an arthropod vector takes a bloodmeal from a viremic vertebrate animal host to maintain the zoonotic cycle. Mosquitoes have been suggested as possible enzootic vectors and/or bridge vectors. Conversely, *Culicoides paraenesis* has been identified as the primary enzootic vector whereby the virus is maintained between humans and midges.

OROV is maintained in a zoonotic cycle (also called the sylvatic cycle) and an enzootic cycle. In the zoonotic cycle, the virus is transmitted between non-human vertebrate animals and associated vectors; while in the enzootic cycle, the virus cycles between human-biting vectors and humans [23,24,25]. When these cycles overlap, a spillover event occurs, spurred either when a human encroaches on the zoonotic cycle or when vector or animals from the sylvatic cycle overlap into the enzootic setting. This occurs through processes such as agricultural expansion or urbanization, or sometimes through a bridge vector that may vacillate between the two settings [26,27]. The primary enzootic vector for OROV has been identified as the biting midge *Culicoides* *paraenesis* [23,28,29,30]. In addition to the primary vector, *Culex quinquefasciatus*, *Cq. venezuelensis*, and *Aedes serratus* are mosquitoes that have been found to transmit OROV but are considered secondary vectors in the enzootic cycle [23,31,32]. Several species of mosquitoes, specifically *Coquillettidia venezuelensis* and *Cx. quinquefasciatus*, have been implicated in maintaining the sylvatic cycle of OROV in non-human mammalian hosts such as non-human primates and sloths, as well as avian species [23,32] (Figure 1).

Before the emergence of Zika and chikungunya in the western hemisphere, OROV was recognized as being the second most common arboviral cause of human febrile illness in Brazil, second to dengue virus [33]. Though this virus is recognized as the etiologic agent of human disease and is known to infect animals, there is a scarcity in screening and detection outside of known areas associated with detected outbreaks [34,35,36]. Nevertheless, there is increased call for expanded screening of OROV across South America due to the significant number of cases and symptom similarity to other arboviruses to accurately attribute disease with specific pathogens [15,35]. However, efficient surveillance of under-detected pathogens requires sufficient data to target efforts either in specific locations or at specific times. The objective of this systematic review (SR) is to characterize the locations where OROV surveillance efforts have either confirmed OROV presence or not, as well as those locations where surveillance studies have detected the OROV vector independent of virus detection.

## 2. Material and Methods

Searches were conducted in August 2020. To assemble the published literature regarding OROV, an online search in the PubMed database was performed using the term “Oropouche”, which returned 143 records. We recognized that perhaps not all relevant publications were indexed in PubMed, so an additional search was performed using Google Scholar using the terms “Oropouche Virus” AND “South America”, which returned 598 records. Because many entomological studies are not indexed in PubMed, we used Google Scholar as the primary source of candidate studies for the search of the primary vector. Thus, to characterize the ecological sites where the primary vector has been studied in South America, a second literature search was performed using Google Scholar with the search terms: “Culicoides paraensis” AND “South America”, which returned 173 records.

### 2.1. Study Selection

Inclusion criteria for the OROV studies were defined as the following: molecular and/or serologic detection, and epidemiological data. Exclusion criteria were defined as the following: reviews, studies without primary data (modeling, assay design, phylogenetics, immunology, physiology, insecticide investigations), duplicate study populations, Oropouche location (not virus), thesis/dissertation, book/book chapter, inaccessible/untranslatable, vector competence studies. The number of records was recorded, and they were included or excluded based on the satisfaction of the defined criteria. Titles were identified and, if obvious, used to exclude studies; abstracts were then read of the remaining studies to determine if exclusion criteria were met; finally, whole papers were read to determine if the studies satisfied inclusion or exclusion criteria.

Inclusion criteria for the arthropod vector of OROV were defined as the following: epidemiological studies and entomological studies including surveillance, field observations, and abundance. Exclusion criteria were defined as the following: reviews, laboratory-based vector competence studies, investigations without primary data, duplicate study populations, thesis/dissertation, book/book chapter, and inaccessible or untranslatable articles. The total number of articles was recorded, and they were either included or excluded based on the satisfaction of the defined criteria. Titles were first examined, followed by abstracts, and then whole paper analysis.

### 2.2. Data Extraction and Synthesis

The records that satisfied inclusion criteria were examined for data extraction and synthesis over all studies. Documented study features included: study date and location, detection of nucleic acid and/or antibodies, laboratory assay performed or vector competence studies, hosts tested and confirmed positive, and ecological and environmental settings. Similarly, the data extracted from *C. paraensis* literature included: surveillance date and location, vector species collected, number of vectors collected, collection methods, and ecological settings. Articles that were returned from the Google Scholar database with the search terms “*Culicoides paraensis*” and “South America” that concentrated on any secondary vectors (*Ae. serratus*, *Cq. venezuelensis*, *Cx. quinquefasciatus*) for OROV were also included. Despite the search criteria targeting the primary vector, several records (n = 14) were returned that focused on secondary vectors. These papers were included in the review.

Variables were identified by descriptive mention in the studies and a master list of variables made. Papers were then reread and tallied for mention of each variable in the list. We considered the presence and absence of the following as indicators of different eco-environmental settings: crops, livestock, military facilities, mining, swamps, coasts, streams, springs, lakes, rivers, dams, restingas (coastal broadleaf forest), floodplains, mangroves, primary forest, secondary forest, cerrado (tropical savanna), and pantanal (tropical wetland). Maps were constructed using QGIS version 3.14.15-Pi [37] and shape files were downloaded from http://tapiquen-sig.jimdofree.com (Carlos Efraín Porto Tapiquén. Geografía, SIG y Cartografía Digital. Valencia, Spain, 2020, accessed on 30 June 2021).

### 2.3. Classification Models

The data were separated into two separate sets: acute (e.g., PCR detected) or convalescent (e.g., antibody) cases. To determine whether any of the eco-environmental variables affected the odds of an acute or past OROV case detection, we built a suite of logistic classification models, employing variable selection methods, and each dataset was independently analyzed. Proportion of people tested that were positive for OROV acute infection, convalescent/past infection was used as the dependent variable and the binary (noted/not mentioned) ecological variables were used as the independent variables. Case studies and case series with total number of patients tested being fewer than 10 were excluded from the statistical analyses from the OROV search dataset. Likewise, to determine associations between eco-environmental variables and the presence of *C. paraensis*, we calculated the proportion of *C. paraensis* found among all insects reported, which was used as the dependent variable and the binary ecological variables were again used as the independent variables. No vector studies were excluded in that corresponding statistical analyses.

The following model variations were considered:

1. All eco-environmental variables were run as independent variables.
2. Reduced models whereby several variables were combined as below and run as a uni-, bi-, and tri-variate models for acute and past infections:
  a. “livestock”, “military”, “crops”, and “mining” were combined into a single “anthropological” variable.
  b. “river”, “dam”, and “pantanal” were combined into a single “water” variable.
  c. “primary” or “secondary” forests were combined into a single “forest” variable.
3. Reduced models whereby the several variables were combined as below and run as a uni-, bi-, and tri-variate models for the presence of the vector:
  a. “livestock”, “military”, and “crops”, were combined into a single “anthropological” variable.
  b. “river”, “dam”, “pantanal”, “swamp”, “streams”, “springs”, and “lakes” were combined into a single “water” variable.
  c. “primary”, “secondary”, “mangrove”, and “restinga” forests were combined into a single “forest” variable.

The *logistf* function was used (package logistf) to fit the models using R Studio (version 1.3.1093) and R version 3.6.3. Significance was assessed at the α = 0.05 confidence level.

### 2.4. Correlation Analyses

For eco-environmental variables, we assigned the value of 1 for present and 0 for absent and calculated the Spearman’s rank correlation coefficient of eco-environmental variables with (1) the proportion of people tested that were positive for OROV acute infection or convalescent/past infection in the PCR and antibody data sets, and (2) the proportion of *C. paraensis* found in the vector data sets. We tested for the significance of each correlation with a two-tailed *t*-test at the α = 0.05 confidence level against the null hypothesis that a correlation was equal to zero.

## 3. Results

**Figure 2:**
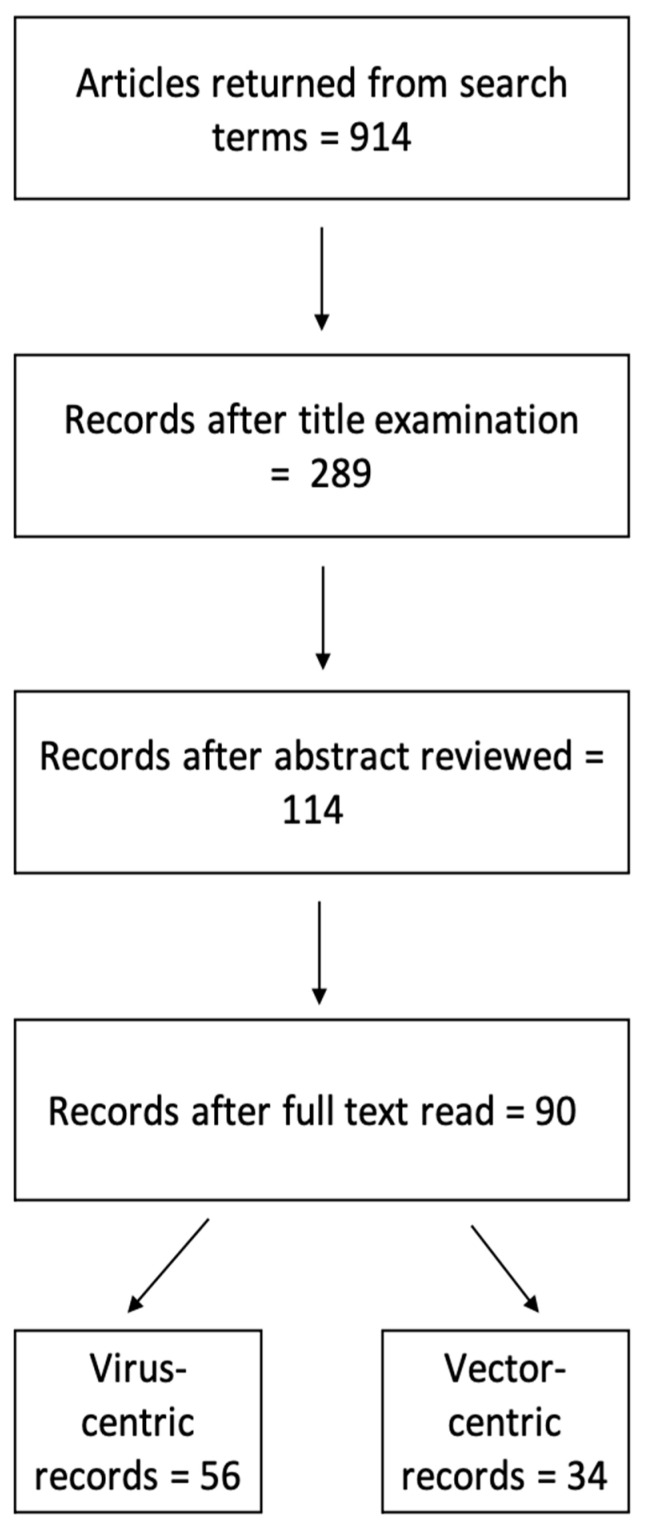
Flow-chart of record processing for inclusion in systematic review.

A literature search in PubMed with the term “Oropouche” returned 143 records. A separate literature search in Google Scholar with the terms “Oropouche virus” AND “South America” returned 598 records. After accounting for duplicate records from the dual database search, a total of 90 articles (only 9.9% of all returned records) satisfied all inclusion criteria. The Google Scholar search regarding the primary arthropod vector for OROV which was carried out using the terms “Culicoides paraensis” and “South America” returned 173 records. Of the 90 articles included, 62.2% involved the investigation of OROV while 37.8% of the studies were focused on entomological surveillance (Figure 2).

### 3.1. OROV Surveillance

**Figure 3:**
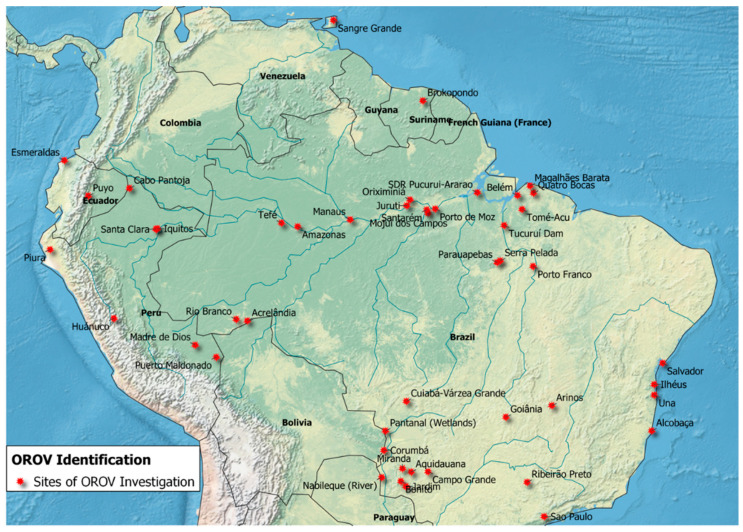
Locations identified where OROV has been detected in South America based on included records.

Across all 56 articles included for OROV surveillance, there were eight separate countries where these investigations took place (Figure 3). There were 37 studies performed in Brazil (63%) [2,5,7,16,17,24,32,38,39,40,41,42,43,44,45,46,47,48,49,50,51,52,53,54,55,56,57,58,59,60,61,62,63,64,65,66,67], followed by 11 studies in Peru (18.6%) [6,18,34,68,69,70,71,72,73,74,75], with the remaining (18.6%) taking place in Ecuador [3,8,15,70,76], Paraguay [70,77], Suriname [78], Trinidad [4], Bolivia [70], and Costa Rica [79].

### 3.2. Arthropod Vector Surveillance

**Figure 4:**
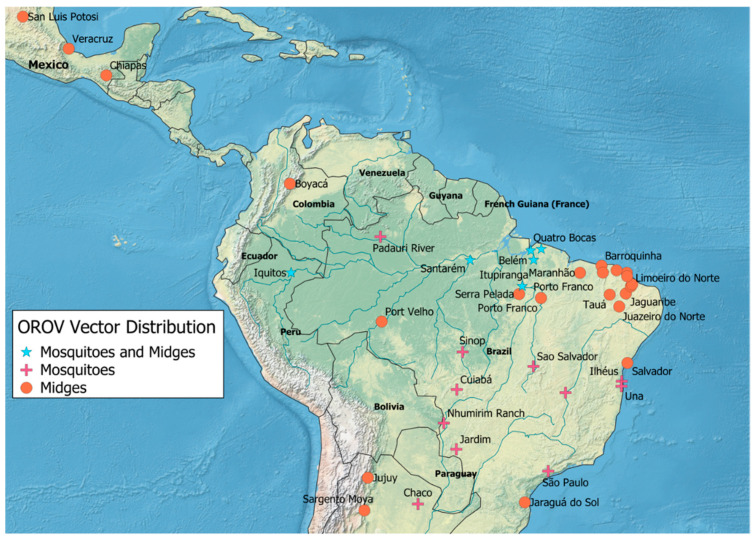
Locations where OROV vectors have been observed in Mexico and South America according to records included in this review. Map generated in ArcGIS.

There were 34 articles that met all inclusion criteria for the *C. paraensis* search. There was a total of seven separate countries in which these surveillance efforts took place in literature included in this review (Figure 4). There were 21 (60%) articles that were based in Brazil [80,81,82,83,84,85,86,87,88,89,90,91,92,93,94,95,96,97,98,99,100], 6 (17.1%) studies in Peru [101,102,103,104,105,106], 4 (11.4%) in Argentina [107,108,109,110], with the remaining (11.4%) taking place in Colombia [30], Paraguay [109], Mexico [111], and Suriname [112].

### 3.3. OROV Detection

OROV detection studies involved both serological and molecular assays across a range of host species. OROV screening was performed on samples from humans, non-human primates, arthropods, mammals, avian species, and reptiles. Of the 56 articles that were focused on the detection of OROV, there were 46 serologic [2,3,5,6,7,8,16,17,18,32,34,38,39,40,41,42,43,45,46,48,49,50,52,53,56,57,58,59,60,62,63,64,65,66,67,68,69,70,71,72,73,74,75,77,78,79], 7 disease etiology investigations where OROV was confirmed [4,15,24,44,47,51,76], and 3 diagnostic development studies [54,55,61].

Several methods were used for the detection of both nucleic acid and antibodies: complement fixation (CF), hemagglutinatin inhibition (HI), neutralization tests, enzyme-linked immunosorbent assay (ELISA), plaque reduction neutralization test (PRNT), indirect immunofluorescence assay (IFA), virus isolation assays, real-time reverse transcription polymerase chain reaction (RT-PCR) and quantitative real-time RT-PCR (qRT-PCR), and Illumina-based sequencing. Of these methods, the most commonly used for antibody detection was ELISA, whereas the most commonly used method for the presence of OROV nucleic acid was RT-PCR.

From the 56 articles included in OROV investigations, 9 (16%) of the total 56 studies found no presence of OROV [43,48,59,62,65,67,75,77,79], whereas 47 (84%) of the studies that screened for OROV found either antibodies to OROV (18 studies) [2,5,18,38,39,40,41,45,46,50,52,58,63,64,68,69,71,78], OROV nucleic acid (16 studies) [3,6,8,15,34,47,49,53,54,55,60,61,66,72,73,76], or both (13 studies) [4,7,16,17,24,32,42,44,51,56,57,65,70]. Humans were tested for OROV antibodies (11 studies) [5,18,38,39,40,58,63,64,68,69,78], OROV nucleic acid (19 of studies) [3,6,8,34,43,47,48,49,53,54,55,59,61,62,66,72,73,76,77], or both (17 studies) [4,7,15,16,17,24,32,41,42,44,51,52,56,57,70,71,74].

Non-human mammals that were tested for OROV include: non-human primates, sloths, avian species, rodents, sheep, reptiles, and horses. There were seven studies that tested non-human primates, five studies tested for antibodies [45,46,50,65,75], one for OROV nucleic acid [60], and one for both [67]. There were three studies that tested samples obtained from sloths where two of these studies tested for antibodies [65,79] and one study tested for OROV nucleic acid [32]. Three studies tested avian samples for antibodies [16,17,58], two studies tested rodents for antibodies [17,58], and one study that tested sheep, caiman, and equine samples for antibodies [2].

## 4. Ecological Factors Associated with OROV Presence

### 4.1. Vector Presence and Diversity

In the 56 studies returned by the search for the virus, several contained information about primary or secondary vectors of OROV. In 8/56 studies (14.3%), surveillance for known OROV vectors was also conducted and presence confirmed of at least one competent species [4,16,17,32,45,49,56,57]. In four of these three studies—three in Para, Brazil [17,49,57] and one in Maranhão, Brazil [56]—in which OROV was found, the presence of *C. paraensis* was documented. Further, 7/56 studies observed mosquito vectors: *Ae. serratus* in four studies located in Trinidad [4] and Brazil (two in Para State [16,32] and one in Mato Grosso do Sul State [45]), and *Cx. quinquefasciatus* in three studies in Brazil (Para State [17,57] and Mato Grosso State [49]). Of the 34 entomology articles, *C. paraensis* was found in 18 studies (52.9%) [30,80,81,90,91,93,94,95,96,97,98,103,104,108,109,110,111,112], and each of the three mosquito vectors were present in seven studies (2.9%), some of which overlapped [82,83,84,85,86,87,88,89,93,101,102,107].

### 4.2. Anthropogenic Land Use

**Figure 5:**
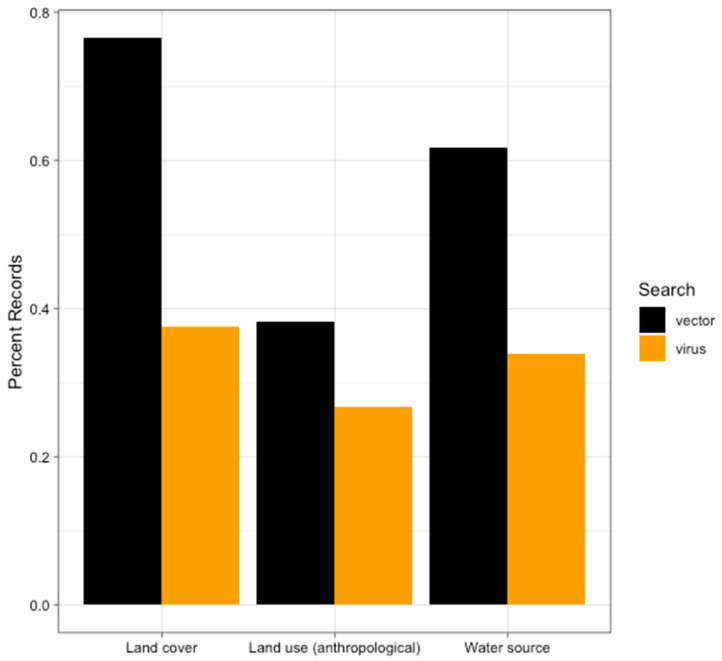
The proportion of records that had observations about land cover, anthropogenic land use, and/or water sources contained within the text according to which search they were returned from. Black represents the proportion of records in the vector data (N = 34) and gold represents the proportion of records in the virus data (N = 56).

For the OROV search, 15/54 (27.8%) articles provided information regarding anthropogenic land use (Figure 5). The uses of land in these locations were crop cultivation, livestock rearing, military outposts, and mining, present in eleven [4,7,16,17,56,57,63,65,68,69,79], six [2,7,17,56,63,79], three [8,18,75], and one [57] studies, respectively. The two most common land uses, crop cultivation and livestock rearing, were both present in five of studies [7,17,56,63,79].

There were 13/34 studies from the vector search that described land use (Figure 5). The most common use of land was crop cultivation (nine studies)) [30,80,85,88,89,104,108,110,112], followed by livestock (seven studies) [80,91,97,99,102,104,108]. In studies, crop cultivation and livestock were noted [80,104,108].

### 4.3. Water Source

In 19 of the 56 articles focusing on OROV surveillance, a nearby water source was noted (Figure 5). In 14 locations there was at least one river present [17,18,38,45,56,58,59,63,67,68,69,71,78,79]. Other sources of water were noted but to a much lesser extent with three locations in the Pantanal (the largest tropical wetland in the world) [2,46,49], two locations in a coastal area [51,55], and two locations near a dam [64,78]. We found that water sources were mentioned in 21 of the 34 articles regarding *C. paraenesis* surveillance (Figure 5). The presence of at least one river was described in 11/21 articles [80,81,82,85,93,94,102,106,109,110,112]. Streams were the second most frequently mentioned in five articles [30,89,90,108,110]. There were four studies that noted marshes/swamps [89,99,107,112], whereas coastal locations [91,94], lakes [83,86,89], floodplains [83,95], and springs [86,89] were all water sources mentioned twice each. There was one location where surveillance took place at the site of a dam [82] and one study that took place in the Pantanal [83].

### 4.4. Land Cover

There were 21 (38.9%) articles that made observations regarding land cover from the 56 studies that focused on OROV surveillance (Figure 5). Locations that have primary or undisturbed forest were most common, mentioned in 16 studies [4,6,8,15,16,17,18,24,59,65,66,67,68,70,72,75]. Secondary forests, or forest locations that had been previously used for anthropogenic purposes were found in two studies [4,69]. A cerrado land cover, which consists of bushy, small bent and twisted trunk trees, was described in four locations [45,46,49,66], while two locations described mountainous terrain [6,57].

Of the 34 studies from the *C. paraensis* search, 26 (76.5%) made note of the land cover (Figure 5). Primary and secondary forest locations were noted in sexteen [30,81,83,85,89,90,95,98,99,102,104,107,108,109,110,112] and eleven studies [80,82,84,86,88,92,99,101,102,104,106], respectively. There were three surveillance studies that took place in locations described as cerrado [83,84,112], while both mangrove [91,112] and restinga land cover [98,100] types were mentioned. Restinga refers to coastal forests with the soil mostly consisting of sand and salt. There were several studies that had overlapping types of land cover which included cerrado, primary forest, and secondary forest locations [83,84,98,99,102,104,112].

### 4.5. Odds of Detecting Acute and Past Infections

Logistic regression run on the full model run with all 12 variables did not reveal any significant predictors for odds of either pcr prevalence (acute infections) or antibody prevalence (past infections). Due to the small dataset and the large number of variables, we next employed a data reduction method and ran the model with uni-, bi-, and trivariate combinations of the variables related to anthropological land-use, the presence of water, and/or forests. In none of the models were there any variables significant for either the acute or past infections. Correlation analysis revealed a single significant correlation between acute infections and the presence of restingas (Spearman’s rho ρ = −0.41, *p*-value = 0.0398). All other correlations were statistically insignificant at the α = 0.05 confidence level.

### 4.6. Odds of Vector Presence

Similarly, the full model of 17 eco-environmental variables for determining differences in odds of detection of the vector species did not reveal any statistically significant predictors for the proportion of *C. paraensis* found. None of the uni-, bi-, or trivariate models run using the data reduction methods returned significant results. This suggests that, in South America, there is still not enough data available to determine whether the detection of *C. paraensis* is likely to be associated with specific eco-environmental variables. Correlation analyses, likewise, found no significant correlations between the proportion of *C. paraensis* found and the eco-environmental variables.

## 5. Discussion

Our primary objective was to assemble the existing data regarding the environmental and ecological factors associated with known detection of OROV in South America, and to identify factors associated with the presence of the primary enzootic vector. Our review of the published data demonstrates that there are similarities among the factors observed in OROV and vector detection. First, the most prominent anthropogenic land use observed was crop cultivation and livestock for both OROV and vector presence. Secondly, rivers were the most common source of water in locations where OROV and arthropod vectors were documented. Lastly, both OROV and vectors were more frequently found in primary forest locations. However, our analyses of these data show that there is no significant signal among these variables and OROV and/or vector presence/absence. This is likely due to a lack of power in the analyses given the relatively small number of records and overall data. Again, this points to the need for further studies to characterize the ecological and environmental drivers of OROV detection and its associated vector(s). Further, more data are needed to determine whether the qualitative patterns detected in the characteristics observed is a result of sampling bias or whether these characteristics truly could be associated with OROV detection.

Because OROV is an arbovirus, the presence of a vector is highly correlated with the likelihood of virus detection [28,29]. Arbovirus transmission potential is measured largely by the vectorial capacity equation, which investigates key variables including such factors as vector density relative to humans, and extrinsic and intrinsic factors that govern the virus–vector dynamics [113]. Often, the processes of transmission within the vector—vector competence and the EIP—are affected by environmental factors, most notably temperature, which can vary in response to anthropogenic land use, land cover, and proximity to water [114,115,116,117]. Therefore, these environmental and ecological characteristics are crucial factors that should be considered in OROV transmission and when planning efforts aimed at detecting this potentially emergent arbovirus.

For example, because a good portion of the *C. paraenesis* lifecycle includes semi-aquatic stages, these arthropods are often found in moist, decaying vegetation such as that from recently harvested farms [117]. *C. paraensis* often lays eggs in moist areas, where developmental stages progress through the lifecycle by feeding on decomposing organic matter, algae, and small vertebrate prey [117]. The necessity of water to the lifecycle of *C. paraensis* (as well as the secondary mosquito vectors) increases the likelihood of finding these arthropods in close proximity to water sources [117]. In addition, water is an attractant to susceptible vertebrate hosts in both uninhabited and inhabited locations, which leads to host–vector interactions necessary for arboviral transmission in the context of potential reservoir hosts, such as avians, non-human primates, and rodents [116]. While *C. paraensis* is known to feed on humans, their opportunistic feeding patterns lead them to feed on other vertebrate hosts in lieu of human presence [117]. In addition, forested environments suitable for reservoir hosts enables arthropod vectors to both acquire and transmit OROV [116,117].

The increasing demand for agricultural and industrial products has resulted in worldwide globalization [118]. The proceeding economic expansion into South America centers around crop production, especially soy, while some areas also take part in the livestock trade [118,119]. In addition, mining for bauxite, nickel, and gold have had a positive impact on economic development in this region [120]. However, these economic gains have had unintended consequences whereby previously uninhabited forests are now in closer proximity to human populations and/or human enterprises. This, in turn, increases the chances of zoonotic spillover events by increasing contact between vector and reservoir vertebrate host populations [26].

There are numerous locations across regions of South America where investigations into the presence of OROV have successfully identified circulation of the virus; however, it is noteworthy that there are geographic gaps in OROV surveillance efforts. These data demonstrate that there was no evidence of surveillance for OROV noted in Colombia, Venezuela, and Guyana, even though all three of these countries border Brazil. Colombia, particularly, borders three countries where OROV has been identified (Figure 3). Further, of the included records from the Google Scholar “*Culicoides paraensis*” and “*South America*” search, entomological surveillance efforts for the primary vector of OROV in South America are predominantly in Brazil, especially on the easternmost portion of the country (Figure 4).

There are obvious gaps in the literature concerning OROV, a neglected tropical, zoonotic disease of emergent public health importance. The most surveillance has been conducted in Brazil (Figure 3). While one *C. paraensis* surveillance study in Colombia verified the presence of the vector, entomological surveillance specific to *C. paraensis* was also found to be primarily concentrated in Brazil, with sporadic surveillance locations in Peru and Argentina [90,103,110]. Additionally, our review provided a preliminary characterization of ecological and environmental factors that may be associated with increased chances of OROV detection and perhaps circulation. However, continued research is needed to fully elucidate OROV transmission dynamics, arthropod vector competence, and OROV distribution to prepare for future emergence or expansion of this public health threat.

## 6. Conclusions

There were notable common ecological aspects associated with both the locations involved in OROV detection and the presence of the vector in the 90 studies included in this systematic review. Ecological characteristics included in both OROV and arthropod vector surveillance studies had crop cultivation as the most common anthropogenic use of land, rivers as the most common water source, and primary forest as the most prominent type of land cover. The observations found within this systematic review illustrate the importance of continued research into the ecological factors associated with the distribution of under-characterized arboviruses. This review reveals the need for continued surveillance of OROV and its arthropod vector, and the description of factors associated with detection may help to prioritize OROV surveillance initiatives.

## Data Availability

Shape files were downloaded from a publically available resource (http://tapiquen-sig.jimdofree.com) and otherwise all data is available within the manuscript.
